# LOCALE: Local-Alignment Embeddings for Noise-Robust DNA Search at SRA Scale*

**DOI:** 10.64898/2026.05.12.724581

**Published:** 2026-05-14

**Authors:** Ryan P. Synk, Prashant Pandey, S. Cenk Sahinalp, Ramani Duraiswami

**Affiliations:** 1 University of Maryland, College Park; 2 Northeastern University, Boston; 3 National Cancer Institute, NIH, Bethesda

## Abstract

Searching petabase-scale repositories of raw sequencing data such as the NIH Sequence Read Archive (SRA) could transform biological discovery, but existing methods either do not scale well or rely on exact k-mer matching that is brittle to sequencing errors and biological divergence. We recast sequence search as dense retrieval: we learn vector embeddings whose inner-product similarity ranks locally aligned sequences above unaligned ones. Our key observation is that effective retrieval does not require accurate regression of global edit distance—it only requires that sequences with better local alignments score higher than sequences with worse ones. We train a DNABERT-2 encoder with an InfoNCE objective on biologically informed augmentations: overlapping crops of parent sequences corrupted with substitutions, insertions, and deletions. On a 50-accession SRA benchmark, LOCALE maintains 62.4% average $\text{Recall}@R_{q}$ at a 10% mutation rate, while every baseline we evaluated falls below 60% $\text{Recall}@R_{q}$ in the noisy-query setting. The advantage holds at scale: on a 500-accession, 15-Gbp benchmark, LOCALE achieves AUPRC 0.508 at 10% mutation versus 0.129 for MetaGraph.

## 1 Introduction

Technologies for sequencing biomolecular data have rapidly decreased in cost [1], leading to explosive growth in available sequence data. The Sequence Read Archive (SRA), a public repository of sequencing data, was at 47 petabytes of data as of 2025 and continues to grow in size by 16% year-on-year [2]. The ability to quickly and accurately search large repositories of raw sequencing data for matches to a query sequence could transform biological discovery. By a *match* we mean a high-identity local alignment: a region of the query that aligns at high identity to a substring of some indexed sequence. To this end, we train a neural network to embed sequences into high-dimensional vectors whose similarity approximates local sequence alignment, enabling large-scale sequence search that is robust to the sequencing errors inherent in raw reads.

*Raw reads* are short biomolecular sequences output directly from the sequencer, and an *accession* is a set of raw reads from the same sequencing run, typically corresponding to one biological sample (see §A.1 for definitions of terms common in sequence Search). Accessions frequently contain tens of millions of reads. With sequence search over these repositories, an epidemiologist studying a new viral strain not yet found in reference databases could quickly search all wastewater accessions to understand the strain’s prevalence; a biologist studying an unidentified “dark matter” sequence could search across all publicly-available reads to find clues to its origin or function.

Existing methods, however, fall short of enabling these large-scale searches. BLAST and other seed-and-extend methods [3, 4] are workhorses of biomolecular sequence search, but they are designed for searching over smaller databases of assembled genomes and do not scale to vast repositories like the SRA. Along with scale, sequencing errors present another challenge. Most SRA records consist of raw, unassembled sequencing reads, and raw long-read data—especially from earlier PacBio and Oxford Nanopore platforms—can contain substantial (> 10%) sequencing errors [5]. These errors can break methods that rely on exactly matching substrings of length-k, known as k-mers, since a single base pair error invalidates an entire k-mer and introduces k erroneous k-mers [6, 7, 8].

While neural network embeddings for biomolecular sequences have been studied [9, 10, 11, 12, 13], they use metric learning to regress the *exact edit distance* on the entire query sequence. However, for biomolecular sequence search, what matters more is *local alignment* (finding the best aligning substrings of two sequences), and not global alignment, which penalizes each mismatch independently and does not capture a partial overlap between the query sequence and the target sequence. A 100 bp match in a 1000 bp sequence, for instance, has an edit distance of 900. Our key observation is that during search, an embedding model does not need to approximate global alignment and the implied edit distance accurately. Instead, it simply needs to rank aligned sequences higher than unaligned ones. Based on this, we employ a DNABERT-2-style [14] encoder with an InfoNCE contrastive objective, using biologically-informed augmentations—overlapping crops of parent sequences corrupted with substitutions, insertions, and deletions—to generate positive pairs. We then demonstrate that our method outperforms competing approaches on real sequencing data.

### 1.1 Contributions:

In summary, we:

- Reformulate sequence search embedding as a partial ranking problem—placing aligned pairs above unaligned ones—rather than regressing edit distance directly.
- Develop a contrastive training pipeline with biologically informed augmentations that yields vector embeddings respecting local alignment.
- Demonstrate improved retrieval over noisy raw-read queries at two benchmark scales and construct an accession-level retrieval benchmark derived from real SRA data following the methodology of MetaGraph [7].

## 2 Related Work

### 2.1 Seed-and-Extend:

Traditional search methods like BLAST [3], and more recent tools like MMseqs2 [4] follow the two-step algorithmic paradigm known as seed-and-extend. First, a fast k-mer matching step finds candidate matches, or seeds. Then the alignment is extended via a dynamic programming step. While widely used for searching databases of reference genomes, these methods were not designed to search over sets of raw reads. These k-mer indexes are flat mappings from k-mers to positions in reads, so query times grow linearly with the size of the indexed database and thus do not scale to SRA-sized repositories. Additionally, these methods do not handle the case where a long query is fractured over many different reads. The reliance on exact matching of k-mer seeds can also fail when a match has been corrupted by sequencing errors. A single mutation changes up to k different k-mers between the original and mutated sequence. For standard k values like 31 [7, 8, 15], this means that a handful of mutations in a 150bp read may eliminate most shared k-mers from the original sequence despite high sequence identity.

### 2.2 Experiment Discovery:

In order to scale searches to billions of raw reads, recent methods like MetaGraph [7] and Mantis [8] have recast search as experiment discovery. The experiment discovery problem instead seeks to return those accessions which contain some user-defined threshold fraction of the query’s k-mers. This is both scalable, consisting of lookups in a mapping of k-mers to accessions, and robust to the fact that searches are performed over sets of unassembled reads. Tools like Mantis and MetaGraph implement this via colored de Bruijn graphs [16], which compress the k-mer-to-accession mapping by exploiting co-occurrence of adjacent k-mers. Despite lower memory costs and faster query times compared to BLAST, these techniques still rely on exact k-mer matching. As with the alignment heuristics explained above, this dependence on exact sequence matching renders these methods highly brittle to mutations and sequence errors between the raw reads and queries. Fig. 2 shows failure mode of k-mer methods and § 4.2 shows that LOCALE is able to maintain high accuracy in this setting.

### 2.3 Deep Learning Methods:

#### 2.3.1 Deep Edit Distance:

A variety of papers have used metric learning to optimize vector embeddings of sequences to approximate the exact string edit distance [9, 10, 11, 12, 13]. These approaches embed sequences into vectors in order to find global, or end-to-end, alignments of sequences. Sequence search, however, requires local rather than global alignments. A *local alignment* between two sequences is the best matching pair of substrings between the two sequences. If a query is much shorter than a target sequence but matches a substring exactly, the edit distance is dominated by length difference. In this case, the edit distance may even be larger than the distance between the query and an unrelated sequence of the same length.

In the case where a query is shorter than a target sequence but contained entirely within it, the exact edit distance may be larger than a low-identity sequence of the same length. Additionally, some methods like Melo-ED and CNN-ED have convolutional architectures requiring fixed-length inputs, preventing comparisons across sequences of different lengths [12, 9]. As shown in §4.2, these edit-distance models can rank globally similar but locally irrelevant sequences above true local matches.

#### 2.3.2 Local Alignment Embeddings:

Recent works like Embed-Search-Align [17] and Neuraligner [18] embed reads in order to align them to an indexed reference genome. In this work, we consider the inverse, where we index all reads across many accessions in order to quickly return matching accessions. Both methods train models primarily on high-identity matches, limiting their applicability to the noisier, more divergent sequences common in raw-read collections. We adapt Embed-Search-Align as a baseline in our experiments by substituting its encoder into our indexing and retrieval pipeline, and show that due to its lack of noise-augmented training its performance decays in the presence of increased mutations (see §4.2).

#### 2.3.3 Dense Retrieval:

In information retrieval, dense retrieval methods [19, 20, 21] learn neural encoders that map queries and documents into a shared embedding space, where relevance is measured by document similarity. Contriever [20], inspired by the momentum contrast [22] trained an encoder without direct supervision via contrastive learning and a memory bank, using augmented views of text spans as positive pairs. Paired with approximate nearest neighbor indexes [23], dense retrieval scales to billions of documents while tolerating lexical variation between queries and matches. These same properties of scale, noise tolerance, and sublinear query time are precisely what experiment discovery over raw reads demands.

## 3 Method

We learn an encoder $f_{\theta }:\Sigma *\to R^{D}$ that maps DNA sequences from Σ={A,G,C,T} into a latent vector space such that the inner product $f_{\theta }(s{)}^{T}f_{\theta }(p)$ ranks locally-aligned sequences s,p above unaligned ones. We first describe our training objective, then our data augmentation strategy, our model architecture and training setup, and finally our search system.

### 3.1 Training Objective

To push aligned sequences together in latent space, we utilize a contrastive representation learning approach with an InfoNCE loss [24]. Given a batch of B positive pairs of aligned sequences ${\left\{\left(q_{i},k_{i}\right)\right\}}_{i=1}^{B}$, we compute the InfoNCE loss with in-batch negatives:

(1) $$ℒ\left(q_{i}\right)=-\text{log}\frac{\text{exp}\left(f_{\theta }{\left(q_{i}\right)}^{T}f_{\theta }\left(k_{i}\right)/\tau \right)}{\text{exp}\left(f_{\theta }{\left(q_{i}\right)}^{T}f_{\theta }\left(k_{i}\right)/\tau \right)+\sum_{j=1,j\neq i}^{B}\text{exp}\left(f_{\theta }{\left(q_{i}\right)}^{T}f_{\theta }\left(k_{j}\right)/\tau \right)},$$

where τ is a temperature hyperparameter that sharpens or smooths the distribution of similarity scores. We set τ=0.05 throughout training. This loss encourages the model to assign high inner-product similarity to positive pairs and low similarity to all other pairs in the batch.

### 3.2 Data Augmentation

**Figure 1:**
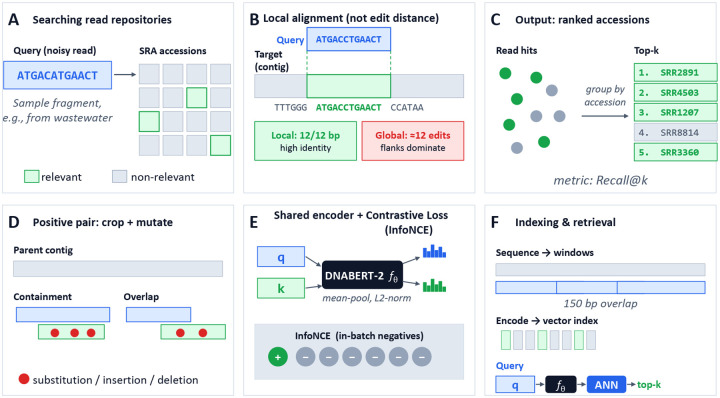
Overview of LOCALE. **(Top row, conceptual.) (A)** A short noisy DNA read (e.g., a wastewater viral fragment) is queried against the NIH Sequence Read Archive (SRA), only some accessions of which are relevant. **(B)** The relevant notion of similarity is *local alignment*: a query may match an internal substring of a target with high identity (green) even when global edit distance is dominated by unmatched flanking regions (red). (C) LOCALE returns a ranked list of *accessions*, obtained by aggregating sequence-level retrieval hits via a per-accession max-similarity rule; we evaluate with accession-level Recall@k. **(Bottom row, methodological.) (D)** Positive pairs (q,k) are constructed by taking containment or overlap crops of a parent contig (a gap-free stretch of DNA assembled from sequencing reads) and corrupting one crop with random substitutions, insertions, and deletions to simulate sequencing error and biological divergence. **(E)** Both crops pass through a shared DNABERT-2 encoder $f_{\theta }$ followed by mean-pooling and $L_{2}$-normalization; we train with an InfoNCE (eq 3.1) objective using simple in-batch negatives, without momentum-contrast machinery. **(F)** At index time, sequences exceeding the encoder context window are split into overlapping windows (150 bp overlap) and embedded; at query time, retrieved hits are grouped by accession and the per-accession score is the maximum inner-product similarity.

**Figure 2:**
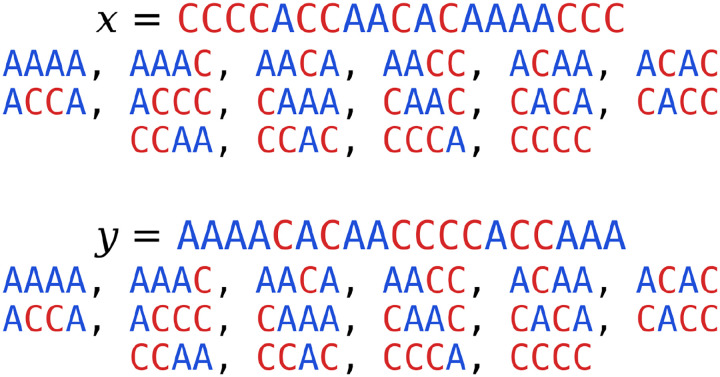
Sequences x and y have the same k-mer representation but low sequence similarity. k-mer-based representations employed in MetaGraph and Mantis are not the right proxy for sequence search since they can introduce a large number of false positives.

We construct positive pairs by taking overlapping crops of a longer parent sequence and corrupting one crop with random mutations, as illustrated in Figure 1.

#### 3.2.1 Cropping:

We test two cropping strategies: *containment* and *overlap*. A containment crop pairs a sequence with a subsequence entirely contained inside it; an overlap crop pairs two sequences that overlap at one end without either being contained in the other. Both are generated from a common parent sequence. We sample crop lengths uniformly between 31 and 256 base pairs. To ensure a sufficiently strong positive match signal, we require that after cropping, the smaller sequence covers at least 40% of the longer one. For overlap crops we further require that the aligned (overlapping) region span at least 40% of the longer crop; for containment crops these two conditions coincide, since the aligned region is exactly the shorter sequence. When matching short reads to longer contigs, as is the case in our benchmark setting, containment crops are especially relevant.

#### 3.2.2 Biologically plausible augmentation:

After cropping, we inject random mutations into one of the two crops to simulate sequencing error and biological divergence. For each pair, we sample a target identity $\rho \in \left[0,\rho_{\text{max}}\right]$ from a Beta distribution—following [25], parameterized by mean and standard deviation rather than the conventional shape parameters α,β, which we find easier to interpret as identity distributions. Given a crop of length L and sampled identity ρ, we apply n=⌊L(1−ρ)⌋ mutations uniformly at random without replacement within the aligned region; each mutation is independently a substitution, insertion, or deletion with probabilities 0.4, 0.3, 0.3. Substitutions choose uniformly among the three non-matching bases; insertions place a uniformly random base before the sampled position. Mutations are applied to only one crop in each pair.

We test four augmentation strengths in our ablations (§ 4.4): *none* (no mutation), *light, medium*, and heavy. Heavy augmentation, which our final model uses, samples target identity from a Beta distribution with mean 80%, $\rho_{\text{max}}=88\%$, and standard deviation 6 percentage points—meaning a typical heavy-augmented training pair has roughly one in five bases differing between query and key. Parameters for light and medium are in the appendix table 4.

### 3.3 Model

We use DNABERT-2 [14] as our encoder backbone and *fine-tune all parameters* (rather than freezing them) under the contrastive objective above. DNABERT-2 uses a byte-pair-encoding tokenizer with a vocabulary size of 4096; for an input sequence of T tokens, the transformer produces contextualized representations $h_{t}\in R^{D}$ per token. We aggregate these to a sequence-level vector by mean pooling over non-padding positions and then $L_{2}$-normalize, constraining representations onto the unit sphere. The encoder has 117M parameters, embedding dimension D=768, and was originally trained on sequences of 700bp.

### 3.4 Training

#### 3.4.1 Data:

We train on two corpora. The *Logan* corpus consists of contigs produced by the Logan project [15], which assembled contigs from every accession in a December 31, 2025 snapshot of the SRA (see species breakdown in appendix table 5). Logan contigs compress raw reads while preserving sequence diversity. We subsample the same 5,000-accession benchmark slice used by [7] down to 50 accessions for training, disjoint from any accessions used for evaluation. The *reference* corpus consists of the whole reference genomes of the unique species within the 50-accession training set available for download on the NCBI Reference Sequence Database [26] (species breakdown in appendix section A.2.4). For training, the reference genomes were chunked into non-overlapping 1024 bp parent sequences. We draw 6 million training pairs from each corpus. Both corpora were used with the same training pipeline and budget; the final reported model uses the Logan corpus, while the comparison to reference-genome training appears in §4.4. Performance between the two models trained on these varying corpora were similar.

#### 3.4.2 Hyperparameters:

We train with AdamW [27] ($\beta_{1}=0.9,\beta_{2}=0.999$, weight decay 10^−2^) at a peak learning rate of 6 × 10^−5^ with a 460-step linear warmup followed by cosine decay. We use a per-device batch size of 64 across 4 nodes of 4 NVIDIA A100 GPUs each, for a batch size of 1024. We train for 5859 steps, requiring approximately 1 hour of wall-clock time (16 A100 hours).

### 3.5 Indexing and Search

#### 3.5.1 Indexing:

Given a set of accessions $𝒰=\left\{A_{1},\ldots ,A_{N}\right\}$, where each accession is set of sequences $\left\{s_{i,1},\ldots ,s_{i,\left|A_{i}\right|}\right\}$ we embed every sequence with the trained encoder to produce a vector index V. Sequences exceeding the encoder’s context window are split into sliding windows with a 150 bp overlap between adjacent windows, and each window is embedded independently.

#### 3.5.2 Search:

Given a query q, we retrieve its top-m nearest sequence embeddings $N_{m}(q)$ from the index by inner-product similarity. We then aggregate this sequence-level shortlist at the accession level: letting $V_{A}$ denote the embeddings of sequences in accession A, the score for A is the maximum similarity over its sequences appearing in the shortlist:

$$s(q,A)=\underset{v\in {𝒩}_{m}(q)\cap V_{A}}{\text{max}}\langle q,v\rangle ,$$

Accessions are returned in descending order of s(q,A). We use m=10 in all experiments. We optionally apply vector quantization methods like RaBitQ [28]; ANN scaling is discussed in §4.3.

## 4 Experiments

### 4.1 Setup

#### 4.1.1 Problem Definition and Metric:

We evaluate LOCALE’s ability to retrieve accessions containing local matches to noisy query sequences—both raw reads and synthetically mutated variants—using a benchmark that we constructed from real SRA data. Given a universe of accessions 𝒰 and query sequence q, we say an accession A is relevant to q if q admits a local alignment to some sequence in A exceeding an identity threshold ι, where identity is defined as the number of exact base pair matches divided by the length of the aligned region. We set ι=0.9 in our experiments. Sequence search methods return a list of accessions ordered by score. We quantify performance with recall:

**Definition 1** (**Recall@k**) *Let* q *be a query sequence and let* ${𝒜}_{q}\subseteq 𝒰$ *its set of relevant accessions, with* $\left|{𝒜}_{q}\right|\geq 1$. *Given a search method that returns a ranked list* $\left(A_{1},A_{2},\ldots \right)$ *of accessions ordered by score, let* ${ℛ}_{k}(q)=\left\{A_{1},\ldots ,A_{k}\right\}$ *be its top*-k *retrievals. Then*

$$\text{Recall}@k(q)=\left|{𝒜}_{q}\cap {ℛ}_{k}(q)\right|/\left|{𝒜}_{q}\right|.$$

Given a batch of queries $Q=\left\{q_{i}\right\}$, the summary metric $\left(\sum_{i}\text{Recall}@\text{k}\left(q_{i}\right))/|Q\right|$, is denoted Average Recall@k. We report two retrieval-quality metrics. Letting $R_{q}=\left|{𝒜}_{q}\right|,\text{Recall}@R_{q}$ evaluates recall at the exact number of relevant accessions for query q, so a perfect retriever achieves $\text{Recall}@R_{q}=1$. We average over queries to obtain Average $\text{Recall}@R_{q}$. We also report the Area Under the Precision-Recall Curve (AUPRC), which aggregates over all k.

#### 4.1.2 Dataset:

We construct a test index over the same accessions as the MetaGraph benchmark [7]–a representative slice of SRA data. For each accession, we index the corresponding contigs from Logan [15], assembled from raw reads. We evaluate at two scales: a 50-accession subset (of which 47 had Logan contigs available at index time, 787 Mbp, 9 million sequences) and a 500-accession subset (500 Logan contigs, 15 Gbp, 138 million sequences). Both subsets are sampled proportionally to the organism distribution of the 100-studies set; details and metadata are in appendix §A.2.2 (tables 6 and 7). No accession is shared between test indexing and model training.

#### 4.1.3 Queries:

##### Query sourcing

Following MetaGraph [7], we draw queries from the raw reads of the same SRA accessions we indexed. This gives a natural ground truth–a raw read is, with high probability, locally alignable to some contig of its source accession–while still posing a non-trivial retrieval problem, since Logan contigs are stitched from many reads and the read constitutes only a local match.

##### Relevance labeling and filtering

To establish ground-truth relevance, we align each query against the contigs of every accession in the index using Minimap2 [29], labeling an accession relevant if any alignment achieves identity ≥ι=0.9. Let ${𝒜}_{q}$ be the resulting relevant set. We discard queries whose origin accession is not in ${𝒜}_{q}$ on the grounds that such reads are not locally alignable to their own source contigs (due, for example, to read-quality or contig-cleaning artifacts) and would penalize all methods. We also exclude queries whose relevant set spans more than 20% of indexed accessions, since these correspond to conserved or repetitive sequences on which even uniformly random rankings achieve high recall. We initially sample 1,000 queries uniformly at random from each raw-read set, apply the filtering above, and randomly subsample to 500 queries; we construct one query set for the 50-accession test and one for the 500-accession test.

##### Synthetic mutation generation

To evaluate retrieval under noise, we generate two mutated variants of each query, with target identities of 95% and 90% (5% and 10% mutation). For a sequence of length L and target identity ρ∈{0.95,0.9}), we sample ⌊L(1−ρ)⌋ positions uniformly without replacement, and at each position we apply a substitution, insertion, or deletion with probabilities 0.4, 0.3, 0.3. Substitutions choose uniformly among the three non-matching bases; insertions place a uniformly random base before the position. Throughout, a mutation rate of 5% or 10% refers to synthetic corruption producing target identities of 95% or 90%, respectively, while relevance of queries to accessions is defined separately using a local-alignment identity threshold of 0.9.

#### 4.1.4 Baselines:

We compare LOCALE to:

- **MetaGraph** [7] a k-mer index method, with accessions scored by k-mer overlap percentage
- **MMseqs2** [4], a seed-and-extend method, with accessions ranked by bit-score alignment to query
- Two vector embedding models, retrieved via the same accession-level aggregation as LOCALE: **LLM-ED** [13], an edit-distance model; and **Embed-Search-Align** [17], a read-to-reference genome mapping model.

For embedding baselines, we substitute their encoder into our indexing and retrieval pipeline (§3.4). MMseqs2 is intractable at the 500-accession scale: the query batch exceeded our 3-hour search-time cutoff. We retain MMseqs2 in the 50-accession comparison because it provides an alignment-based reference point for a high-quality but less-scalable search strategy.

#### 4.1.5 Hardware:

We measure search performance on a single node with one AMD EPYC 7763 64-core CPU, four NVIDIA A100 (Ampere) 40GB GPUs, and 256GB of DDR4 DRAM. Vector-index construction parallelizes across 4 nodes for embedding, but search runs on a single node. MetaGraph is CPU-only; MMseqs2 does not support GPU nucleotide search.

### 4.2 Retrieval Quality

**Figure 3:**
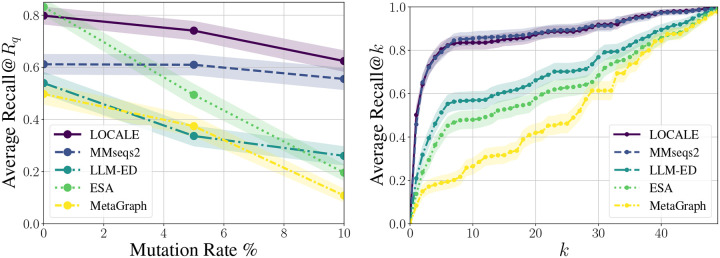
Left: Average $\text{Recall}@R_{q}$ over the 50-accession dataset. Each point shows average recall across 500 queries - recall is defined in 4.1.1. Mutation rates of 0%, 5% and 10% correspond to query identities of 100%, 95% and 90% respectively. Right: Average Recall@k for increasing values of returned accessions, k - out of 50, under 10% mutation rate. Our method achieves high recall under noise, while k-mer methods and other vector embeddings degrade sharply at 10% mutation rates.

Figure 3 reports Average $\text{Recall}@R_{q}$ as a function of mutation rate (left panel) and Recall@k for varying k at the 10% mutation rate (right panel), both on the 50-accession dataset. LOCALE maintains 62.4% Average $\text{Recall}@R_{q}$ at the 10% mutation rate, while every baseline drops below 60%, with the gap widening as noise increases.

The Embed-Search-Align (ESA) baseline slightly outperforms LOCALE in the noiseless setting (83% vs. 80%), but its performance degrades sharply as mutation increases, consistent with its lack of heavy noise-augmented training. LLM-ED, which regresses exact edit distance, trails LOCALE even in the noiseless case (54% vs. 80%), consistent with our argument that exact edit-distance regression is the wrong objective for retrieval, where what matters is ranking aligned sequences above unaligned ones.

MetaGraph illustrates a ranking failure mode distinct from pure sensitivity loss: in the noiseless setting it achieves high Recall@7 (96.6%, see appendix table 8 for Recall@7 data) but low $\text{Recall}@R_{q}$ (49.8%), indicating that relevant accessions are often found but are not ranked consistently ahead of irrelevant ones. This could be due to spurious k-mer overlap from non-aligned sequences causing the method to rank irrelevant accessions highly. Under noise, k-mer overlap to relevant accessions decreases sharply, dropping its Average $\text{Recall}@R_{q}$ to only 10.8% at the 10% mutation rate.

As Table 1 confirms, the AUPRC ordering matches the recall ordering: LOCALE is highest under noise (0.700 at 10%), while ESA leads in the noiseless case (0.876) but collapses to 0.284 at 10%; MetaGraph degrades fastest (0.579 to 0.139). Notably, LOCALE’s AUPRC at 10% mutation (0.7) exceeds MMseqs2 at any mutation rate.

The timing column of Table 1 adds a complementary picture. MMseqs2, the alignment gold standard, needs 384.7 s to batch-search 500 queries against the 50-accession index—two to three orders of magnitude slower than every embedding method, and intractable at the 500-accession scale we evaluate in §4.3. LOCALE in float32 runs in 1.86 s, and LOCALE+RaBitQ in 0.60 s—a 641× speedup over MMseqs2 while *exceeding* its AUPRC at the 10% mutation rate (0.700 vs. 0.656). MetaGraph is the fastest at 0.125 s, reflecting the advantage of hash-table k-mer lookup over either alignment or vector search; LOCALE+RaBitQ is 5× slower than MetaGraph but achieves six times its AUPRC under noise (0.700 vs. 0.139), positioning LOCALE as the noise-robust point on the latency–accuracy frontier.

### 4.3 Scalability

We test LOCALE’s ability to scale by indexing the 500-accession subset. At approximately 160M total vectors, a full-precision (float32) index would occupy a prohibitive 490 GB. Quantization techniques such as RaBitQ [28] achieve 1-bit quantization for high-dimensional vectors at little loss in retrieval accuracy; we apply 1-bit RaBitQ quantization and evaluate its effect on both datasets.

Table 2 compares LOCALE+RaBitQ to MetaGraph at both scales. We do not run MMseqs2 at the 500-accession scale, as it exceeded a pre-set 3-hour limit on the query batch. The other vector-embedding baselines are also omitted from the 500-accession evaluation: at 50 accessions LOCALE outperforms all vector-based baselines (Figure 3, Table 1), so we treat LOCALE as the representative of the embedding family at scale and reserve the head-to-head comparison for MetaGraph, which represents a fundamentally different (k-mer) approach.

Quantization substantially reduces memory with only modest loss in retrieval quality. On the 50-accession benchmark, 1-bit RaBitQ reduces $\text{Recall}@R_{q}$ at 10% mutation from 62.4% to 58.0% while shrinking the index dramatically; at 500 accessions, the quantized index occupies 15.85 GB and still substantially outperforms MetaGraph under noise. Compared to MetaGraph at the same 500-accession scale, LOCALE+RaBitQ is roughly 4× larger in memory but achieves nearly 4× higher $\text{Recall}@R_{q}$ under 10% mutation (47.2% vs. 12.0%) and nearly 4× higher AUPRC (0.508 vs. 0.129). The timing comparison is intrinsically asymmetri—LOCALE uses GPU dot-product search while MetaGraph is CPU-only—and we report it for completeness rather than as a head-to-head claim.

Beyond quantization, the dense-retrieval framing opens a path to further scaling through approximate nearest-neighbor (ANN) techniques. Graph-based indexes [30, 31, 32] and inverted-file indices [33] offer sublinear query times at high recall. Future work can integrate these into LOCALE to scale to substantially larger accession counts at bounded latency.

### 4.4 Ablation Studies

Table 3 reports single-axis ablations from our baseline configuration. Cropping strategy is the most consequential design choice. Containment-only cropping outperforms overlap-only and the combined strategy by 14.0 and 9.5 percentage points, respectively, with the gap widening as eval noise increases. We attribute this to the asymmetric query-target length distribution containment produces, which better matches our downstream alignment setting where short reads are aligned against longer references.

**Training on Logan contigs matches reference-genome training** (62.4 vs. 61.2 at 10%), indicating that assembled metagenomic contigs provide sufficient sequence diversity for contrastive pretraining despite their lower curation. This is practically useful, as Logan offers orders of magnitude more sequence than curated references.

**Training mutation strength controls noise robustness at no clean-data cost.** Recall at 10% eval mutation rises monotonically from 27.7 (no training mutations) to 61.2 (heavy), a gain of 33 points, while clean-eval recall remains within a 1.5-point band across all four settings. Aggressive mutation augmentation leaves clean-query performance essentially unchanged while substantially improving noisy-query recall.

## 5 Conclusion

We presented LOCALE, a contrastive embedding model for DNA sequence search that recasts retrieval over raw-read repositories as a dense retrieval problem rather than as edit-distance regression. Our key observation is that retrieval requires only that aligned pairs score higher than unaligned ones, not that distances be approximated accurately. Building on this, we trained a DNABERT-2 encoder with an InfoNCE objective on biologically informed augmentations—containment and overlap crops of parent contigs corrupted with substitutions, insertions, and deletions. On a 50-accession SRA benchmark, LOCALE maintains 62.4% Average $\text{Recall}@R_{q}$ at a 10% mutation rate while every baseline—including k-mer indexes, alignment tools, and prior DNA embedding methods—falls below 60%, with the gap widening as noise increases. The same advantage holds at scale: on a 500-accession, 15-billion-base-pair index, LOCALE reaches an AUPRC of 0.508 at 10% mutation while MetaGraph, the only baseline tractable at this scale, reaches 0.129. With 1-bit RaBitQ quantization the index compresses to 15.85GB at a cost of only 4.4 percentage points of $\text{Recall}@R_{q}$, showing learned embeddings can be made memory-competitive with k-mer indexes while remaining substantially more robust to noise.

Upon publication we will release the trained LOCALE encoder, the training and evaluation code, and the constructed retrieval benchmark—including the SRA accession lists, the queries with their minimap2-derived relevance labels, and the mutation-injection scripts—to support reproduction and to provide the community with a noise-robust retrieval benchmark over real SRA data.

Beyond the immediate results, we view LOCALE as evidence that the retrieval-quality bottleneck for petabase-scale sequence search is no longer scalability alone but *noise tolerance* at scale. Casting sequence search as dense retrieval opens a path to leveraging the substantial body of work on approximate nearest-neighbor indexing, quantization, and graph-based search developed in the information retrieval and vector-database communities, and we expect future gains to come from integrating these techniques rather than from larger encoders alone.

## 6 Limitations

### 6.1 Index size:

LOCALE’s vector index is larger than the k-mer index of MetaGraph in absolute terms: at 50 accessions, the full-precision index occupies 0.94GB versus MetaGraph’s 0.31 GB, and at 500 accessions, the full precision index would occupy 490GB versus MetaGraph’s 3.72 GB. The 1-bit RaBitQ quantization variant reduces this to 15.85GB at 500 accessions while losing only 4.4 percentage points of $\text{Recall}@R_{q}$, putting LOCALE within an order of magnitude of k-mer index sizes, but further compression—via product quantization, learned codebooks, or distilled encoders with smaller embedding dimensions—may be necessary for deployment at full SRA scale.

### 6.2 Encoder constraints:

Four constraints of our current encoder limit applicability. First, sequences exceeding the DNABERT-2 context window must be split into overlapping windows and embedded independently, which discards long-range structure within a single sequence; longer-context DNA encoders would remove this restriction. Second, our model does not handle reverse complements natively: a query and its reverse complement currently produce different embeddings, so deployment requires either querying both strands or canonicalizing sequences at index time. We did not evaluate this trade-off, and a strand-equivariant encoder is a natural direction for future work. Third, all our queries are short reads (100–300 bp); performance on longer queries (kilobase-scale, e.g., full genes or viral segments) would require chunking the query and aggregating across chunk-level matches, which we have not evaluated. Lastly, our encoder is fine-tuned from a base model: training a larger encoder model from scratch could increase embedding performance.

### 6.3 Evaluation scope:

We evaluate on two scales (50 and 500 accessions) drawn from a single benchmark slice of the SRA, originally constructed by [7]. While this slice is representative of SRA composition, broader generalization—across sequencing platforms, taxonomic distributions, and read-length regimes—remains to be tested. Mutation rates above 10% (corresponding to query identity below 90%) are not evaluated; queries with extreme divergence from the indexed targets, such as those arising from distantly related organisms or heavily error-prone older long-read data, may degrade performance further than the trends we report would predict.

## 7 Broader impacts:

LOCALE accelerates search over publicly available sequencing data and could enable applications such as faster pathogen surveillance, viral variant tracking, and the characterization of biological “dark matter” sequences in metagenomic samples. Because the released artifact is an embedding encoder for retrieval over already-public sequences—not a generative model and not a model trained on private or restricted data—we consider the dual-use risk to be no greater than that of existing widely deployed sequence search tools such as BLAST. We will release the model and benchmark with documentation describing intended use and known limitations.

## Figures and Tables

**Figure 4:**
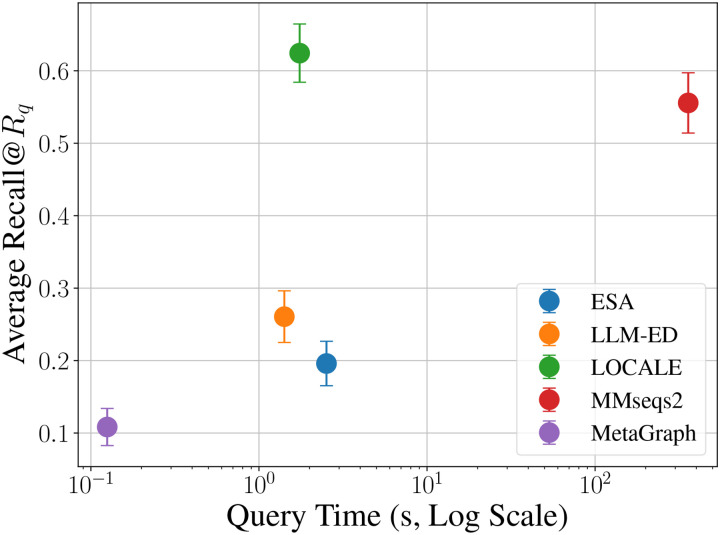
$\text{Recall}@R_{q}$ vs. query time (log scale) on 50 acc dataset with 10% mutations; upper-left is better. LOCALE achieves high recall at low search times, while MetaGraph’s speed comes at the cost of recall.

**Table 1:** AUPRC and search latency over the 50-accession dataset (10% mutation queries, 500 queries batched, averaged over 4 runs with warm start). MetaGraph and MMseqs2 run on CPU; vector methods on GPU. MMseqs2 does not support GPU nucleotide search.

Method	AUPRC			Latency
	0%	5%	10%	(s)
**LOCALE+RaBitQ**	0.852	0.791	0.671	0.603
**LOCALE**	**0.856**	**0.801**	**0.700**	1.86
MMseqs2	0.691	0.709	0.656	384.70
ESA	0.876	0.588	0.284	2.52
LLM-ED	0.633	0.452	0.359	1.42
MetaGraph	0.579	0.404	0.139	**0.125**

**Table 2:** Index characteristics: MetaGraph vs. LOCALE+RaBitQ at two accuracy operating points.

	50 acc (787 Mbp)		500 acc (15 Gbp)	
	MetaGraph	LOCALE	MetaGraph	LOCALE
Indexed units	k-mers	vectors	k-mers	vectors
Index size (GB)	**0.309**	0.941	**3.72**	15.85
Query latency* (ms/read)	**0.16**	0.603	**0.26**	5.15
*Avg $\text{Recall}@R_{q}$*				
0% Mutations	0.498±0.040	**0.791**±0.033	0.455±0.034	**0.725**±0.034
5% Mutations	0.375±0.039	**0.723**±0.037	0.330±0.034	**0.650**±0.037
10% Mutations	0.108±0.025	**0.580**±0.041	0.120±0.024	**0.472**±0.038
*AUPRC*				
0% Mutations	0.579±0.025	**0.852**±0.025	0.497±0.025	**0.761**±0.031
5% Mutations	0.404±0.029	**0.791**±0.029	0.342±0.027	**0.680**±0.030
10% Mutations	0.139±0.022	**0.671**±0.034	0.129±0.022	**0.508**±0.037

Mbp, megabase pairs; Gbp, gigabase pairs. Best value per operating point in **bold**.

* Query latency on different hardware (MetaGraph CPU; LOCALE GPU).

**Table 3:** Ablations. $\text{Recall}@R_{q}$ at three evaluation mutation rates.

Configuration	0%	5%	10%
**Baseline** (Heavy / Contain. / Logan)	79.9±0.033	74.1±0.036	62.4±0.040
Cropping: Overlap	76.3±0.035	65.5±0.040	47.2±0.042
Cropping: Both	79.5±0.033	68.5±0.038	51.7±0.041
Training data: Reference	80.1±0.033	74.1±0.036	61.2±0.040
Training mutation: None	79.6±0.033	62.8±0.040	27.7±0.037
Training mutation: Light	81.1±0.032	74.6±0.036	57.8±0.041
Training mutation: Medium	79.7±0.033	71.4±0.038	60.0±0.041

**Baseline**: heavy training mutations, containment cropping, Logan training data. Each row below baseline changes one axis.

## A Appendix A

### A. 1 Glossary of Biological Terms

- **Raw read:** A raw read is a sequence fragment produced directly by a sequencing instrument before assembly, and typically before substantial error correction or polishing.
- **Contig:** A contig is a contiguous stretch of DNA sequence without gaps, assembled from overlapping sequencing reads using direct sequence information.
- **Accession:** An accession, or accession number, is a unique identifier assigned to a database record or unit of molecular data, such as a sequence, sample, study, or run. For the purposes of this paper, we use the term “accession” to refer to a single sequencing run.
- **SRA:** The Sequence Read Archive (SRA) is NCBI’s public repository for high-throughput sequencing data, storing raw sequencing reads and related alignment information.
- **Logan:** Logan is a large-scale assembled sequence resource built from SRA data that transforms short reads into longer contigs.
- **Local Alignment:** Local alignment is the alignment of subsequences to identify the highest-scoring region of similarity between two biological sequences rather than forcing an end-to-end match.
- **Edit distance:** Edit distance is the minimum number of insertions, deletions, and substitutions required to transform one sequence or string into another.
- k-**mer:** A k-mer is a subsequence of length k extracted from a longer biological sequence such as a read, contig, or genome.

### A. 2 Data and Training

#### A.2.1 Augmentation Parameters

Parameters for light, medium, heavy augmentation distributions: To simulate errors in sequences at training time, we sample identities from a Beta distribution parametrized by a mean and standard deviation rather than conventional shape parameters α,η (following the methodology of [25]). Table 4 lists the three augmentation configurations we used in our experiments:

**Table 4:** Augmentation configurations used in our experiments. Sequence identities are sampled from a Beta distribution reparametrized by mean, standard deviation, and maximum (following Wick [25]) rather than by the conventional shape parameters α,β. Values are reported as percent identity.

Configuration	Mean (%)	Std. dev. (%)	Max (%)
Light	95.0	2.5	99.0
Medium	90.0	6	98.0
Heavy	80.0	6	88.0

#### A.2.2 Logan Data

Table 5 describes the species breakdown of the Logan training dataset. Tables 6 and 7 describe the 50 and 500 acc tests sets, respectively. Each table contains the number of accessions from the respective species, most sets have majority mouse and human data, consistent with their status as the most heavily represented species in the SRA [15]. The tables list the total number of base pairs and total number of contigs across all accessions from the species. They also list n50, which is a metric for identifying sequence length.

All accessions were selected as subsets from the roughly 5,000-accession 100-studies experiment from [7]. Training and testing accession sets were ensured to be exclusive.

To construct evaluation subsets, a target number of accessions are drawn by first sampling organisms with probability proportional to their frequency in the full 100-study pool of [7], then selecting one accession uniformly at random from that organism’s candidates. This procedure ensures that the organism composition of each subset mirrors that of the broader collection, capturing the taxonomic diversity of the original benchmark set.

**Table 5:** Training set composition of Logan training data.

Organism	Num. Acc	Total bp	Total Num Seqs	Median n50 (min-max)
Mus musculus	15	413.66 Mbp	3855960	151.0 (118–468)
Homo sapiens	9	1.67 Gbp	9305243	289.0 (35–483)
seawater metagenome	4	2.00 Mbp	54863	32.0 (32–57)
Danio rerio	3	19.72 Mbp	130283	515.0 (310–546)
Arachis hypogaea	3	1.29 Gbp	17309343	120.0 (102–124)
soil metagenome	2	121.75 kbp	2492	131.5 (43–220)
tree metagenome	2	13.81 kbp	85	221.0 (203–239)
Bacillus anthracis	2	10.95 Mbp	2413	31327.5 (30498–32157)
Clarkia pulchella	2	53.09 kbp	409	153.5 (153–154)
Allium cepa	1	43.64 Mbp	178830	421.0 (421–421)
Pisum sativum	1	133.33 Mbp	2425608	54.0 (54–54)
Pilobolus umbonatus	1	240.26 kbp	2290	218.0 (218–218)
Aspergillus nidulans	1	32.37 Mbp	83061	45279.0 (45279–45279)
Glycera tridactyla	1	29.03 Mbp	147084	352.0 (352–352)
sludge metagenome	1	31.32 kbp	803	34.0 (34–34)
mouse gut metagenome	1	13.98 kbp	348	36.0 (36–36)
rhizosphere metagenome	1	5.20 kbp	48	182.0 (182–182)

**Table 6:** 50 accession test set composition

Organism	Num. Acc	Total bp	Total Num Seqs	Median n50 (min-max)
Mus musculus	17	417.31 Mbp	4328280	151.0 (40–151)
Bacillus anthracis	7	37.23 Mbp	7896	32158.0 (30936–36724)
Homo sapiens	6	62.46 Mbp	639179	201.0 (34–246)
Clarkia pulchella	4	295.07 kbp	2163	154.0 (153–154)
tree metagenome	3	168.45 kbp	4051	36.0 (36–60)
Danio rerio	3	7.20 Mbp	50104	589.0 (276–597)
Aedes albopictus	2	0 bp	0	None (None–None)
Pisum sativum	2	176.70 Mbp	3473131	47.5 (46–49)
soil metagenome	1	25.76 kbp	221	181.0 (181–181)
rhizosphere metagenome	1	2.01 kbp	22	177.0 (177–177)
Phoebe zhennan	1	83.85 Mbp	359828	426.0 (426–426)
seawater metagenome	1	146.44 kbp	3280	39.0 (39–39)
human metagenome	1	2.60 Mbp	71265	31.0 (31–31)
mouse gut metagenome	1	0 bp	0	None (None–None)

**Table 7:** 500 accession test set composition.

Organism	Num. Acc	Total bp	Total Num Seqs	Median n50 (min-max)
Mus musculus	208	5.83 Gbp	55628210	151.0 (32–890)
Homo sapiens	102	5.52 Gbp	43695426	235.0 (33–1080)
Bacillus anthracis	59	324.07 Mbp	84721	32158.0 (3086–39145)
Danio rerio	40	182.02 Mbp	1110290	462.0 (58–683)
human metagenome	18	786.97 Mbp	2416685	610.5 (31–9642)
Ips typographus	17	613.21 Mbp	3586454	501.0 (234–849)
Pisum sativum	16	1.44 Gbp	28297461	48.0 (43–51)
seawater metagenome	7	10.97 Mbp	307004	32.0 (31–32)
Oncorhynchus mykiss	4	138.35 Mbp	815756	320.0 (312–325)
SARS-CoV-2	4	14.23 Mbp	247637	85.5 (46–156)
Apis mellifera	4	86.95 Mbp	307545	630.5 (613–677)
Salmo salar	3	77.94 Mbp	261946	4839.0 (4329–5632)
Verticillium longisporum	2	28.18 Mbp	83834	857.5 (812–903)
Glycera tridactyla	2	54.10 Mbp	318198	226.0 (95–357)
Pilobolus umbonatus	2	9.63 Mbp	160632	58.0 (57–59)
Drosophila melanogaster	2	45.60 Mbp	76602	1417.0 (1408–1426)
Drosophila simulans	1	23.30 Mbp	36457	1533.0 (1533–1533)
Aspergillus nidulans	1	29.94 Mbp	10340	55596.0 (55596–55596)
Pediculus humanus	1	22.79 Mbp	74020	1046.0 (1046–1046)
Glycera lapidum	1	30.03 Mbp	403636	123.0 (123–123)
Tetragenococcus koreensis	1	2.65 Mbp	1398	22976.0 (22976–22976)
coal metagenome	1	1.54 Mbp	43921	31.0 (31–31)
Heliconius erato hydara	1	1.39 Mbp	13457	189.0 (189–189)
Pseudomonas putida	1	4.71 Mbp	28646	841.0 (841–841)
Kordia sp. TARA_039_SRF	1	8.87 Mbp	190990	37.0 (37–37)
Xanthomonas arboricola	1	5.34 Mbp	7806	15212.0 (15212–15212)

#### A.2.3 Test Dataset Accessions

Information about the min and max base pair cutoffs for indexing and the sampling strategy

#### A.2.4 Reference Genome Training Dataset

Of the accessions in the Logan training data 5, there were 17 total “organisms”. Of those, 6 were metagenomic accessions, leaving 11 species. 7 of those 11 species had genomes available for download on RefSeq, and they were downloaded to make up the reference sequence dataset. These collectively contained a total of 13 billion base pairs, which after chunking into 1024bp chunks left a total of 13.3M rows. These were randomly sampled down to 6 million to match the number of logan rows.

**Table:**

Species	Accession	Total Base Pairs
Homo sapiens	GCF_000001405.40	3099441038
Mus musculus	GCF_000001635.27	2728206152
Bacillus anthracis str. ’Ames Ancestor’	GCF_000008445.1	5503926
Aspergillus nidulans FGSC A4	GCF_000011425.1	29828291
Arachis hypogaea	GCF_003086295.3	2557413415
Lathyrus oleraceus	GCF_024323335.1	3796066963
Danio rerio	GCF_049306965.1	1448791966

#### A.3 Full Experimental Results

Tables 8 and 9 give the full experimental results for the 50 and 500 accession experiments.

**Table 8:** Retrieval performance on the 50-accession dataset for raw read queries across all methods and mutation rates.

Mutation rate	Method	R@7	$\text{R}@R_{q}$	AUPRC
0.00	LOCALE	0.946±0.018	0.799±0.033	0.856±0.024
	MMseqs2	**0.989±0.008**	0.612±0.039	0.691±0.025
	MetaGraph	0.967±0.014	0.498±0.039	0.579±0.025
	LLM-ED	0.792±0.033	0.540±0.042	0.633±0.035
	ESA	0.945±0.019	**0.832±0.030**	**0.876±0.023**
0.05	LOCALE	0.901±0.025	**0.741±0.036**	**0.801±0.029**
	MMseqs2	**0.976**±**0.012**	0.610±0.039	0.709±0.026
	MetaGraph	0.683±0.040	0.375±0.039	0.404±0.029
	LLM-ED	0.678±0.038	0.337±0.039	0.452±0.033
	ESA	0.746±0.036	0.495±0.041	0.588±0.035
0.10	LOCALE	0.831±0.031	**0.624±0.040**	**0.700**±**0.034**
	MMseqs2	**0.845**±0.031	0.556±0.041	0.642±0.032
	MetaGraph	0.202±0.035	0.108±0.026	0.139±0.022
	LLM-ED	0.563±0.041	0.261±0.036	0.359±0.033
	ESA	0.467±0.041	0.196±0.031	0.284±0.029

$R_{q}$ denotes the number of ground-truth accessions for query q. Best result per (mutation rate, metric) shown in **bold**

**Table 9:** Retrieval performance on the 500-accession dataset for raw read queries across all mutation rates.

Mutation rate	Method	R@10	$\text{R}@R_{q}$	AUPRC
0.00	LOCALE+RaBitQ	0.837±0.028	**0.725**±0.035	**0.761**±0.031
	MetaGraph	**0.870**±0.023	0.455±0.034	0.497±0.025
0.05	LOCALE+RaBitQ	**0.757**±0.033	**0.650**±0.037	**0.680**±0.034
	MetaGraph	0.664±0.037	0.330±0.034	0.342±0.027
0.10	LOCALE+RaBitQ	**0.614**±0.038	**0.472**±0.038	**0.508**±0.037
	MetaGraph	0.250±0.036	0.120±0.024	0.129±0.022

$R_{q}$ denotes the number of ground-truth accessions for query q. Best result per (mutation rate, metric) shown in **bold**.

##### A.3.1 Training Compute

Final training runs for a single model ran for approximately 1 hour on 16 A100 GPUs for a total of 16 A100 hours. The approximate total compute used (tracked on a cluster account) totaled 961 node hours allocated, or 3844 A100 hours.
